# TonEBP Suppresses the HO-1 Gene by Blocking Recruitment of Nrf2 to Its Promoter

**DOI:** 10.3389/fimmu.2019.00850

**Published:** 2019-04-18

**Authors:** Eun Jin Yoo, Hwan Hee Lee, Byeong Jin Ye, Jun Ho Lee, Chae Young Lee, Hyun Je Kang, Gyu Won Jeong, Hyun Park, Sun Woo Lim, Whaseon Lee-Kwon, Hyug Moo Kwon, Soo Youn Choi

**Affiliations:** ^1^School of Life Sciences, Ulsan National Institute of Science and Technology, Ulsan, South Korea; ^2^Transplantation Research Center, Catholic University of Korea, Seoul, South Korea

**Keywords:** NFAT5, M1 macrophages, M2 macrophages, inflammation, innate immunity

## Abstract

TonEBP is a key transcriptional activator in macrophages with an M1 phenotype. High expression of TonEBP is associated with many inflammatory diseases. Heme oxygenase-1 (HO-1), a stress-inducible protein, is induced by various oxidative and inflammatory signals, and its expression is regarded as an adaptive cellular response to inflammation and oxidative injury. Here, we show that TonEBP suppresses expression of HO-1 by blocking Nrf2 binding to the HO-1 promoter, thereby inducing polarization of macrophages to the M1 phenotype. Inhibition of HO-1 expression or activity significantly reduced the inhibitory responses on M1 phenotype and stimulatory effects on M2 phenotype by TonEBP knockdown. Additional experiments showed that HO-1 plays a role in the paracrine anti-inflammatory effects of TonEBP knockdown in macrophages. Identification of HO-1 as a downstream effector of TonEBP provides new possibilities for improved therapeutic approaches to inflammatory diseases.

## Introduction

Macrophages are a heterogeneous population of immune cells that is present in all tissues and plays a central role in initiation and resolution of inflammation induced by pathogens or tissue damage (1, 2). Macrophages can acquire two distinct functional phenotypes, classical (M1) and alternative (M2), depending on the activating (environmental) stimulus (3, 4). Whereas, the M1 phenotype plays a causal role in inflammatory diseases, the M2 phenotype functions to resolve pathologic inflammation and aid tissue repair during wound healing (5, 6). Plasticity and flexibility are key features of activated macrophages (5–7). Macrophages can undergo dynamic transition between the M1 and M2 states and promote differentiation of neighboring macrophages to their same activation state. Moreover, dynamic changes in macrophage phenotype frequently reveal divergent roles in health and disease. Thus, identification of molecules and mechanisms associated with phenotypic switching of macrophages provides a molecular basis for macrophage-centered diagnostic and therapeutic strategies.

Heme oxygenase (HO) is the rate-limiting enzyme during heme degradation (8), which leads to generation of carbon monoxide (CO), free iron, and biliverdin (9–11). These by-products of HO enzymatic activity are regarded as cytoprotective molecules because of their antioxidant activity [reviewed in (12, 13)]. Two mammalian HO isoforms, HO-1 and HO-2, have been identified (13). HO-1 is a stress-inducible protein induced by various oxidative and inflammatory signals, while HO-2 is a constitutively expressed form. HO-1 has strong immunomodulatory and anti-inflammatory properties (14), which have been demonstrated in HO-1-deficient mice and human cases of genetic HO-1 deficiency (15–20). At present, evidence suggests that induction of HO-1 can drive the phenotypic shift from M1 to M2 in macrophages [(21), reviewed in (22, 23)] HO-1 modulates the immune system during homeostasis and disease by regulating the function and phenotype of macrophages (21, 24–26).

Tonicity-responsive enhancer binding protein (TonEBP), also known as nuclear factor of activated T cells 5 (NFAT5), belongs to the Rel family of transcriptional factors, which includes nuclear factor-κB (NF-κB) and NFAT1-4 (27, 28). TonEBP was initially identified as the central regulator of cellular responses to hypertonic stress (27, 29–31). Recent studies show that high expression of TonEBP in humans and mice is associated with inflammatory and autoimmune diseases (32–36). TonEBP induces M1 macrophages by stimulating expression of pro-inflammatory genes and by suppressing expression of anti-inflammatory genes (37–39). Consequently, downregulation of TonEBP reduces inflammation, thereby helping to prevent inflammatory and autoimmune diseases (32–36). Here, we explored the potential interplay between TonEBP and HO-1 in macrophages. We found that TonEBP is a potent suppressor of HO-1 in human and mouse macrophages. Double knockdown of TonEBP/HO-1 or co-treatment with a HO inhibitor reduced the inhibitory responses on M1 phenotype and stimulatory effects on M2 phenotype by TonEBP knockdown, thereby supporting a role of HO-1 in the anti-inflammatory effects of TonEBP knockdown in macrophages.

## Material and Methods

### Animals, Peritoneal Macrophages, and Bone Marrow-Derived Macrophages

The TonEBP^+/Δ^ mice on C57BL/6 background (31) were crossed back to the C57BL/6 line (The Jackson Laboratory, Bar Harbor, ME) to produce TonEBP^+/Δ^ animals and their TonEBP^+/+^ littermates. Mice were kept on a 12-h light/dark cycle with free access to standard chow and water. Peritoneal macrophages (PMs) were isolated from our previously developed mouse model of type 1 diabetes (35). Briefly, males were selected and made diabetic by daily intraperitoneal injections of freshly prepared streptozotocin (STZ) (50 mg/kg body weight; Sigma-Aldrich, St. Louis, MO) in 0.1 M citrate buffer (pH 4.5) for 4 days. Animals displaying fasting blood glucose levels above 250 mg/dl after 2 weeks of STZ injections were considered diabetic. Control, non-diabetic animals were injected with the buffer. Six weeks post the STZ injections the animals were analyzed for PMs. PMs were isolated from non-diabetic and diabetic mice as described (40). In short, 1 ml thioglycollate (30 mg/ml) was injected intraperitoneally and the peritoneal cells were collected 4 days later. The macrophages were adhesion-purified for 1 h followed by a wash with PBS to remove non-adherent cells and analyzed. Bone marrow cells obtained from femurs were differentiated for 7 days using 20% L929-conditioned medium, as a source of M-CSF, to obtain bone marrow-derived macrophages (BMDMs) (41). The cells were treated as indicated in the figure legends and analyzed. All experimental protocols were approved by the Institutional Animal Care and Use Committee of the Ulsan National Institute of Science and Technology (UNISTACUC-12-15-A).

### Isolation of the Human Primary Monocytes and Differentiation of Monocyte-Derived Macrophages

Human monocyte-derived macrophages were prepared as described previously (39). The study was approved by the Institutional Review Board of the Ulsan National Institute of Science and Technology (UNISTIRB-15-25-A). Briefly, human peripheral blood mononuclear cells (PBMCs) were isolated by centrifugation of whole blood (donated by healthy volunteers) on Histopaque-1077 (Sigma-Aldrich, St. Louis, MO, USA). Monocytes were enriched from freshly isolated PBMCs by positive selection on CD14 microbeads followed by separation on MACS columns (Miltenyi Biotec, Bergisch, Germany). Macrophages were obtained from human monocytes after 7 days of culture in RPMI-1640 medium supplemented with 10% fetal bovine serum (FBS), 1% sodium pyruvate, 0.1% β-mercaptoethanol, and human M-CSF (20 ng/ml; Miltenyi Biotec) (42).

### Cell Culture, Transfection, and Adenoviral Infection

Human monocyte-like THP-1 (ATCC TIB-202) cells were cultured in Dulbecco's Modified Eagle's Medium (DMEM) containing 10% FBS (ThermoFisher Scientific Inc., Waltham, MA, USA) and penicillin/streptomycin (100 U/ml and 100 μg/ml, respectively; GE Healthcare Life Sciences, UT, USA) and then differentiated into macrophages by exposure to 5 ng/ml phorbol 12-myristate 13-acetate (PMA; Sigma-Aldrich) for 2 days. The murine macrophage cell line RAW264.7 (ATCC TIB-71) was cultured in DMEM containing 10% FBS and penicillin/streptomycin (100 U/ml and 100 μg/ml, respectively). All siRNA duplexes were purchased from Integrated DNA Technologies (Coralville, IA, USA). Human monocyte-derived macrophages and PMA-differentiated THP-1 and RAW264.7 cells were transfected with concentration-matched pairs of scrambled (Scr) siRNA or with siRNAs specific for target genes at concentration of 2 nM using HiPerFect transfectant (Qiagen, Valencia, CA, USA) as previously described (42) or using lipofectamine 2000 (Invitrogen, Carlsbad, CA, USA) according to the manufacturer's instructions, respectively, for 24 h. The transfected cells were then cultured in fresh complete medium, treated with vehicle or chemicals and analyzed as indicated in the figure legends. For overexpression, RAW264.7 cells were infected with an empty control virus (Ad-EV) or an adenovirus carrying the human TonEBP gene (Ad-TonEBP) at a multiplicities of infection (MOI) of 50 for 24 h followed by treatment with LPS (100 ng/ml) for 6 h. The 4 kb fragment of the mouse HO-1 promoter (−4,045/+74 pGL2), a gift from Dr. S.W. Chung (University of Ulsan, Ulsan, South Korea) (43), was subcloned into pGL3B (Promega, Madison, WI, USA). AREs or TonE sites in the promoter were mutated using a two-step PCR procedure and overlapping internal primers. All plasmids were purified using an endotoxin-free purification system (Qiagen) and transfected into cells using lipofectamine 2000 (Invitrogen).

### Immunoblot Assay

Western blotting was performed using standard methods. Briefly, cells were washed with cold PBS and lysed in RIPA buffer [10 mM Tris (pH 7.5), 150 mM NaCl, 1 mM EDTA, 1 mM EGTA, 1% Triton X-100] containing 1 mM sodium orthovanadate, phosphatase inhibitor cocktail, and protease inhibitor cocktail. Lysates were centrifuged at 16,000 × g for 15 min at 4°C. The protein concentration was measured in a BCA protein assay system (Pierce, Rockford, IL, USA). Proteins were resolved by SDS-PAGE, transferred to nitrocellulose membranes (Whatman, Clifton, NJ, USA), and probed with anti-TonEBP (26), anti-HO-1, anti-HO-2, anti-p65, anti-lamin B (all from Santa Cruz Biotechnology, Santa Cruz, CA, USA), anti-Nrf2 (Abcam, Cambridge, UK), and anti-Hsc70 (Rockland, Gilbertsville, PA, USA) antibodies.

### RNA Isolation and qPCR

Total RNA was isolated from human monocyte-derived macrophages and cultured cells using TRIzol reagent (Invitrogen). First-strand cDNA was synthesized with 2 μg of total RNA and subjected to quantitative real-time PCR (qPCR) using SYBR Green mastermix in a LightCycler 480 system (Roche, Rotkreuz, Switzerland). Relative amount of mRNA was determined by using the comparative CT (ΔΔCT) method, normalized to cyclophilin A gene as the internal control and expressed as arbitrary units. Primers used are described in Supplementary Table 1.

### Immunocytochemistry

The cells were grown on glass coverslips and fixed with 4% paraformaldehyde in PBS (pH 7.4) for 20 min at 4°C. Cells were permeabilized with 0.25% Triton-X 100 in PBS for 30 min and blocked with PBS containing 5% FBS and 5% bovine serum albumin for 1 h at room temperature. After incubation with rabbit anti-Nrf2 overnight at 4°C, the cells were washed with PBS and treated with goat anti-rabbit Alexa Fluor 488-conjugated secondary antibodies for 1 h. Cells were washed with PBS and incubated in 0.1 μg/ml Hochest (DAPI) for 30 min. After wash with PBS, coverslips were mounted onto microscope slides. Images were recorded using an Olympus FV1000 confocal fluorescence microscope.

### ROS Assay

Cells transfected with Scr siRNA or siRNA targeting TonEBP were pre-treated for 30 min with vehicle or NAC (10 mM) and then cultured in the presence of LPS (100 ng/ml). Then, cells were trypsinized and resuspended in PBS. Intracellular accumulation of ROS was measured using a flow cytometer (Becton-Dickinson, Franklin Lakes, NJ, USA) and the fluorescent probe 2′,7′-dichlorodihydrofluorescein diacetate (Sigma-Aldrich).

### Luciferase Reporter Assay

Cells were transfected for 48 h with Scr siRNA or siRNA targeting TonEBP, followed by transfection with the HO-1 promoter-driven luciferase reporter vector. The Renilla luciferase reporter plasmid was used as a control for transfection efficiency. At 24 h post-transfection, cells were treated with LPS (100 ng/ml). After 8 h, cells were lysed in passive lysis buffer and a luciferase assay was performed using the dual-luciferase reporter system (Promega).

### ChIP Assay

Chromatin immunoprecipitation (ChIP) was performed using a commercial kit (Millipore, Bedford, MA, USA). In brief, cells were crosslinked with formaldehyde (1% final concentration; Sigma-Aldrich) followed by addition of 125 mM glycine. After washing, chromatin fragmentation was performed by sonication on ice to yield an average fragment length <500 bp. Supernatants containing fragmented lysates were diluted 10-fold with chromatin dilution buffer. Samples were pre-cleared for 1 h at 4°C with protein A Sepharose beads (Millipore, MA, USA) that were pre-adsorbed with salmon sperm DNA. Specific antibodies (anti-Nrf2 IgG, anti-Pol II IgG, normal rabbit IgG (Abcam), anti-TonEBP serum, and normal rabbit serum (Merck Millipore, Darmstadt, Germany) were added after removing the pre-clearing beads. After adding the antibodies, the lysates were incubated overnight at 4°C. After elution and reverse crosslinking the antibody/DNA complexes, DNA was purified using a DNA purification kit (Qiagen) and analyzed by qPCR using primer pairs covering AREs, TonE, or TSS regions of the HO-1 promoter and exon 3 of the HO-1 gene. Primers used for qPCR are described in Supplementary Table 1. Immunoprecipitated DNA from each sample was normalized to its respective chromatin input.

### Transwell Co-culture Assay

BMDMs were plated in 6-well plates (Corning Incorporated, Corning, NY, USA). RAW264.7 cells were plated on transwell permeable supports with 0.4 μm pore size (Corning Incorporated), transfected with Scr siRNA or siRNA specific for target genes for 24 h, and treated with LPS (100 ng/ml) for 12 h. The cells were then added to 6-well companion plates containing the BMDMs and co-cultured for 3 or 12 h. At the end point of the experiment, BMDMs were collected for use in a gene expression assay to assess the paracrine effects of macrophages.

### Statistical Analysis

Data are expressed as the mean + SD or SEM. Statistical significance was estimated using two-way ANOVA with Tukey's *post-hoc* test for multiple comparisons. All statistical analyses were performed using GraphPad Prism 5.0 software (GraphPad, CA, USA).

## Results

### TonEBP Suppresses Expression of HO-1 in Macrophages

**Figure 1:**
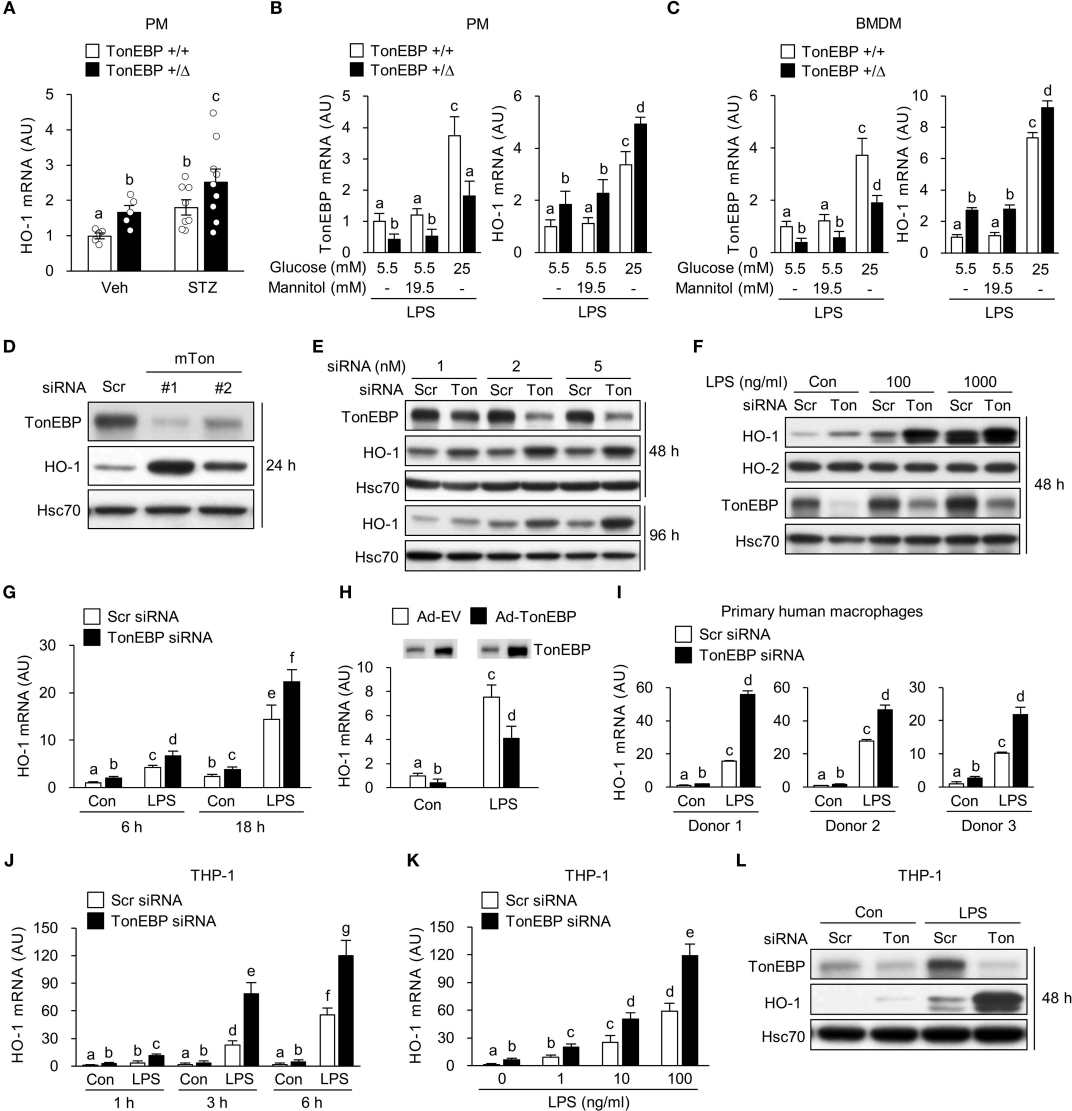
TonEBP reduces expression of HO-1 both in human and murine macrophages. **(A)** Peritoneal macrophages (PM) were obtained from non-diabetic (Veh, *n* = 5) and streptozotocin-induced diabetic (STZ, *n* = 8–9) TonEBP^+/Δ^ and TonEBP^+/+^ mice (34). The abundance of HO-1 mRNA was measured by quantitative RT-PCR. Mean + SEM. **(B,C)** PM **(B)** and bone marrow-derived macrophages (BMDM) **(C)** obtained from TonEBP^+/+^ or TonEBP^+/Δ^ mice were cultured in normal glucose (5.5 mM), high glucose (25 mM), or 5.5 mM glucose + 19.5 mM mannitol (osmotic control for high glucose) for 24 h and then treated with LPS (100 ng/ml) for 6 h. Quantitative RT-PCR was performed to measure expression of mRNA encoding TonEBP and HO-1. **(D)** RAW264.7 cells were transfected with scrambled [Scr (-)] or two siRNAs (Ton #1 or Ton #2) targeting different regions of mouse TonEBP mRNA for 24 h. Immunoblotting to detect TonEBP, HO-1 and Hsc70 was performed. **(E–G)** RAW264.7 cells transfected with scrambled (Scr) siRNA or siRNA targeting TonEBP (Ton) for 24 h. **(E)** Transfected cells were further cultured for 24 or 72 h, followed by immunoblotting to detect TonEBP, HO-1, and Hsc70. **(F)** Transfected cells were treated with vehicle (Con) or LPS (100 or 1,000 ng/ml) for 24 h and immunoblotted with antibodies specific for TonEBP, HO-1, HO-2, and Hsc70. **(G)** Transfected cells were treated with LPS (100 ng/ml) for 6 or 18 h, and abundance of HO-1 mRNA was measured by quantitative RT-PCR. **(H)** RAW264.7 cells infected with adenovirus expressing TonEBP (Ad-TonEBP) or with empty vector (Ad-EV) at an MOI of 50 for 24 h and then treated with LPS for 6 h, followed by immunoblotting to detect TonEBP and quantitative RT-PCR to detect HO-1 mRNA. **(I)** Human peripheral blood monocyte-derived macrophages were transfected for 48 h with Scr siRNA or siRNA targeting TonEBP and then treated with LPS (100 ng/ml) for 6 h. Quantitative RT-PCR to measure HO-1 mRNA was performed. **(J,L)** Human PMA-differentiated THP-1 cells transfected for 24 h with Scr siRNA or siRNA targeting TonEBP. **(J,K)** Transfected cells were treated with vehicle (Con) or LPS as indicated. The abundance of mRNA encoding HO-1 was measured by quantitative RT-PCR. **(L)** Transfected cells were treated with vehicle (Con) or LPS (100 ng/ml) for 24 h, followed by immunoblotting to detect TonEBP, HO-1, and Hsc70. **(A–C,G–K)** Two-way ANOVA with Tukey's *post-hoc* test was used for multiple comparisons. Different letters indicate statistical differences at *P* < 0.05. **(B,C,G–K)** Data (mean + SD) were from three independent experiments (*n* = 3) each with more than three replicates. **(D–F,L)** Data are representative of three independent experiments. AU, arbitrary units.

We previously reported that TonEBP in macrophages promotes hyperglycemia-mediated proinflammatory activation and chronic renal inflammation leading to diabetic nephropathy (DN) (35). Given the protective role of HO-1 on diabetic complications including DN (24, 44, 45), we asked whether TonEBP affected HO-1 expression in macrophages. To address the question, we examined peritoneal macrophages (PMs) obtained from our previously developed mouse model of type 1 diabetes (35). In macrophages from both diabetic and non-diabetic animals, TonEBP haplo-deficiency (TonEBP^+/Δ^) was associated with elevated HO-1 mRNA expression (Figure 1A). In order to characterize the regulation of HO-1 by TonEBP further, we examined PMs and bone marrow derived macrophages (BMDMs) obtained from non-diabetic TonEBP^+/+^ and TonEBP^+/Δ^ mice. PMs and BMDMs were cultured with medium containing normal (5.5 mM) or high (25 mM) glucose in the presence or absence of lipopolysaccharide (LPS), a potent trigger of hyperglycemia-induced inflammation and diabetic complications (23, 46, 47), to mimic a diabetic condition. PMs and BMDMs from the TonEBP^+/Δ^ mice cultured in normal glucose (5.5 mM) showed reduced TonEBP expression and enhanced HO-1 expression compared to those from TonEBP^+/+^ littermates in both resting and LPS-stimulated cells (Supplementary Figure 1A). Raising glucose concentration to 25 mM in the presence of LPS resulted in a higher expression of TonEBP and HO-1 mRNA in PMs (Figure 1B) and BMDMs (Figure 1C) while addition of mannitol to the same osmolality did not. Importantly, the cells from the TonEBP^+/Δ^ mice showed enhanced HO-1 expression compared to those from TonEBP^+/+^ littermates. These data suggest that elevated levels of TonEBP may limit hyperglycemia-mediated induction of HO-1 in macrophages.

Next, we asked whether knocking down TonEBP by siRNA-mediated gene silencing would affect expression of HO-1. RAW264.7 cells were transiently transfected with two siRNAs (mTon #1 and mTon #2) targeting different regions of the mouse TonEBP mRNA. Both siRNAs efficiently reduced protein levels of TonEBP and increased expression of HO-1 protein after 24 h of transfection (Figure 1D). Targeting TonEBP by siRNA mTon #1 resulted in a dose-dependent knockdown (Figure 1E). This led to increased expression of HO-1 protein for up to 96 h (Figure 1E). For the following experiments, we used the siRNA mTon #1 at 2 nM, because siRNA mTon #1 was more effective in silencing TonEBP than mTon #2 (Figure 1D). LPS increased expression of HO-1 (43) and TonEBP (37, 39) proteins (Figure 1F), as previously reported. Notably, TonEBP knockdown increased expression of HO-1 protein and mRNA in resting and LPS-stimulated RAW264.7 cells (Figures 1F,G). Neither LPS nor TonEBP knockdown affected expression of the HO-2 protein, a constitutive isoform (Figure 1F). High glucose (25mM) enhanced the expression of both TonEBP and HO-1 mRNA in response to LPS in RAW264.7 cells, and TonEBP knockdown increased the expression of HO-1 mRNA both under normal and high glucose conditions (Supplementary Figure 1B). We found that adenoviral vectors can be used to transduce RAW264.7 cells without toxicity up to MOI of 100 without cytotoxicity (Supplementary Figure 1C). Overexpression of TonEBP using the adenoviral vector at an MOI of 50 resulted in a reduced expression of HO-1 mRNA in resting and LPS-stimulated cells (Figure 1H), further confirming that TonEBP suppresses HO-1 expression in murine macrophages.

We asked whether the suppression of HO-1 by TonEBP occurred in human macrophages. For this we used human monocyte-derived macrophages obtained from three donors as described previously (39) and macrophages differentiated from the human monocyte cell line THP-1. LPS induced expression of HO-1 mRNA in human monocyte-derived macrophages, and TonEBP knockdown increased the expression of HO-1 mRNA under unstimulated and LPS-stimulated conditions (Figure 1I). Similar results were observed for macrophage-differentiated THP-1 cells. Expression of HO-1 in response to LPS was induced at 1 h, and increased further up to 6 h (Figure 1J). This response was dose-dependent (Figure 1K). TonEBP knockdown increased expression of HO-1 in THP-1 cells under unstimulated and LPS-stimulated conditions (Figures 1J,K). The elevated HO-1 mRNA levels were associated with increased expression of HO-1 protein (Figure 1L). These data demonstrate that TonEBP suppresses HO-1 expression in human and murine macrophages.

### TonEBP Induces the Macrophage M1 Phenotype via Downregulation of HO-1

**Figure 2:**
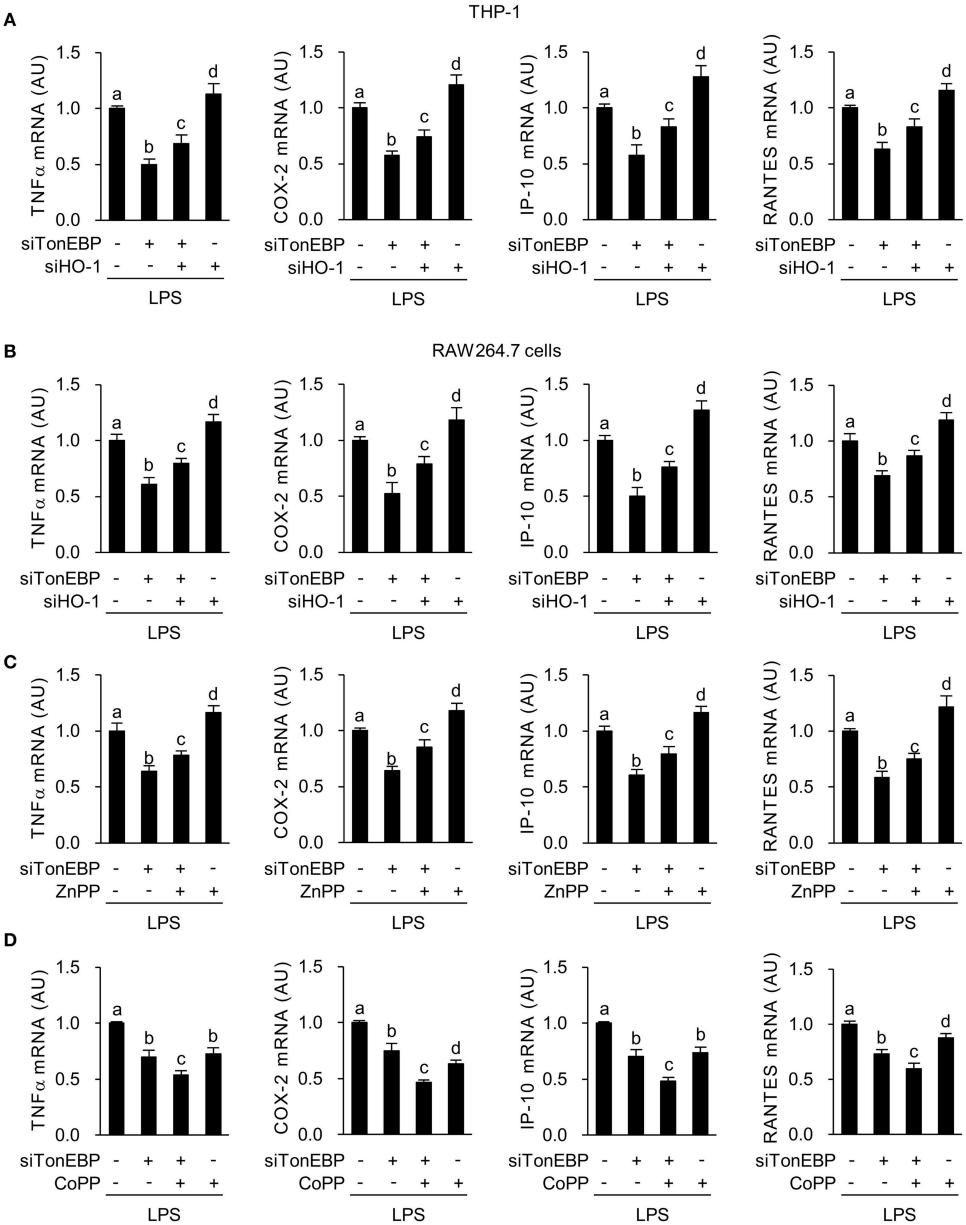
TonEBP induces the macrophage M1 phenotype by downregulating HO-1. **(A,B)** Differentiated THP-1 **(A)** and RAW264.7 **(B)** cells were transfected with scrambled [Scr (-)], TonEBP-targeting, and HO-targeting siRNA in the combinations indicated at the bottom of the panels for 24 h. The concentration of total siRNA was equalized by adjusting the concentration of Scr (-) siRNA. Transfected cells were then treated with LPS (100 ng/ml) for 3 h (for TNFα) or 6 h (for COX-2, IP-10 and RANTES). Expression of mRNA was measured by quantitative RT-PCR. **(C,D)** RAW264.7 cells transfected with Scr (-) or TonEBP-targeting siRNA were treated for 3 h (for TNFα) or 6 h (COX-2, IP-10, and RNATES) with LPS in the presence of ZnPP (20 μM), CoPP (5 μM), or vehicle (-). Expression of mRNA was measured by quantitative RT-PCR. Two-way ANOVA with Tukey's *post-hoc* test was used for multiple comparisons. Different letters indicate statistical differences at *P* < 0.05. Data (mean + SD) were from three independent experiments (*n* = 3) each with more than three replicates. AU, arbitrary units.

Here we asked whether M1 genes tumor necrosis factor α (TNFα), cyclooxygenase-2 (COX-2), chemokine (C-X-C motif) ligand 10 (IP-10), and chemokine (C-C motif) ligand 5 (RANTES), that are associated with diabetic complications (48–50), were affected by the increased expression of HO-1 in response to TonEBP knockdown. First, we examined the effects of siRNA-mediated silencing of TonEBP and HO-1 in PMA-differentiated THP-1 and RAW264.7 cells. Both siRNAs (hTon #1 and hTon #2) targeting different regions of the human TonEBP mRNA efficiently reduced protein levels of TonEBP and increased expression of HO-1 protein after 24 h of transfection in THP-1 cells (Supplementary Figure 1D). For the following studies we used the siRNA hTon #1 at concentration of 2 nM. TonEBP targeting siRNA TonEBP reduced expression of TonEBP mRNA while increasing HO-1 mRNA in THP-1 (Supplementary Figure 2A) and RAW264.7 cells (Supplementary Figure 2B), whereas siRNA targeting of HO-1 reduced HO-1 mRNA without affecting TonEBP mRNA expression. LPS induces rapid expression of the pro-inflammatory M1 genes (51). LPS-induced expression of mRNA encoding TNFα, COX-2, IP-10, and RANTES in THP-1 cells fell after TonEBP knockdown. Notably, TonEBP/HO-1 double knockdown reduced the suppressive effects of TonEBP knockdown on expression of these genes (Figure 2A). Same pattern of changes was observed in RAW264.7 cells (Figure 2B), demonstrating that TonEBP induces M1 genes in human and murine macrophages (at least in part) by downregulating HO-1.

Next, we examined the effects of zinc protoporphyrin (ZnPP), which inhibits HO-1 activity and cobalt protoporphyrin (CoPP), an inducer of HO-1. Both ZnPP and CoPP increased HO-1 mRNA expression, a finding in line with previous reports (52, 53) while not affecting TonEBP mRNA expression (Supplementary Figures 2C,D). TonEBP knockdown significantly increased CoPP or ZnPP-mediated expression of HO-1 mRNA (Supplementary Figures 2C,D). ZnPP increased expression of TNFα, COX-2, IP-10, and RANTES in LPS-stimulated RAW264.7 cells (Figure 2C). In addition, inhibition of these genes' expression upon TonEBP knockdown was attenuated by treatment with ZnPP (Figure 2C). CoPP increased expression of HO-1 protein in resting and LPS-stimulated RAW264.7 cells and TonEBP knockdown increased CoPP-mediated protein expression of HO-1 (Supplementary Figure 2E). CoPP reduced expression of TNFα, COX-2, IP-10 and RANTES in LPS-stimulated RAW264.7 cells (Figure 2D). Furthermore, treatment of TonEBP knockdown cells with CoPP exacerbated the reduction in these genes' mRNA expression induced by TonEBP knockdown (Figure 2D). The opposite actions of ZnPP and CoPP provide further support that M1 induction by TonEBP is mediated by downregulation of HO-1.

### TonEBP Suppresses the Macrophage M2 Phenotype via Downregulation of HO-1

**Figure 3:**
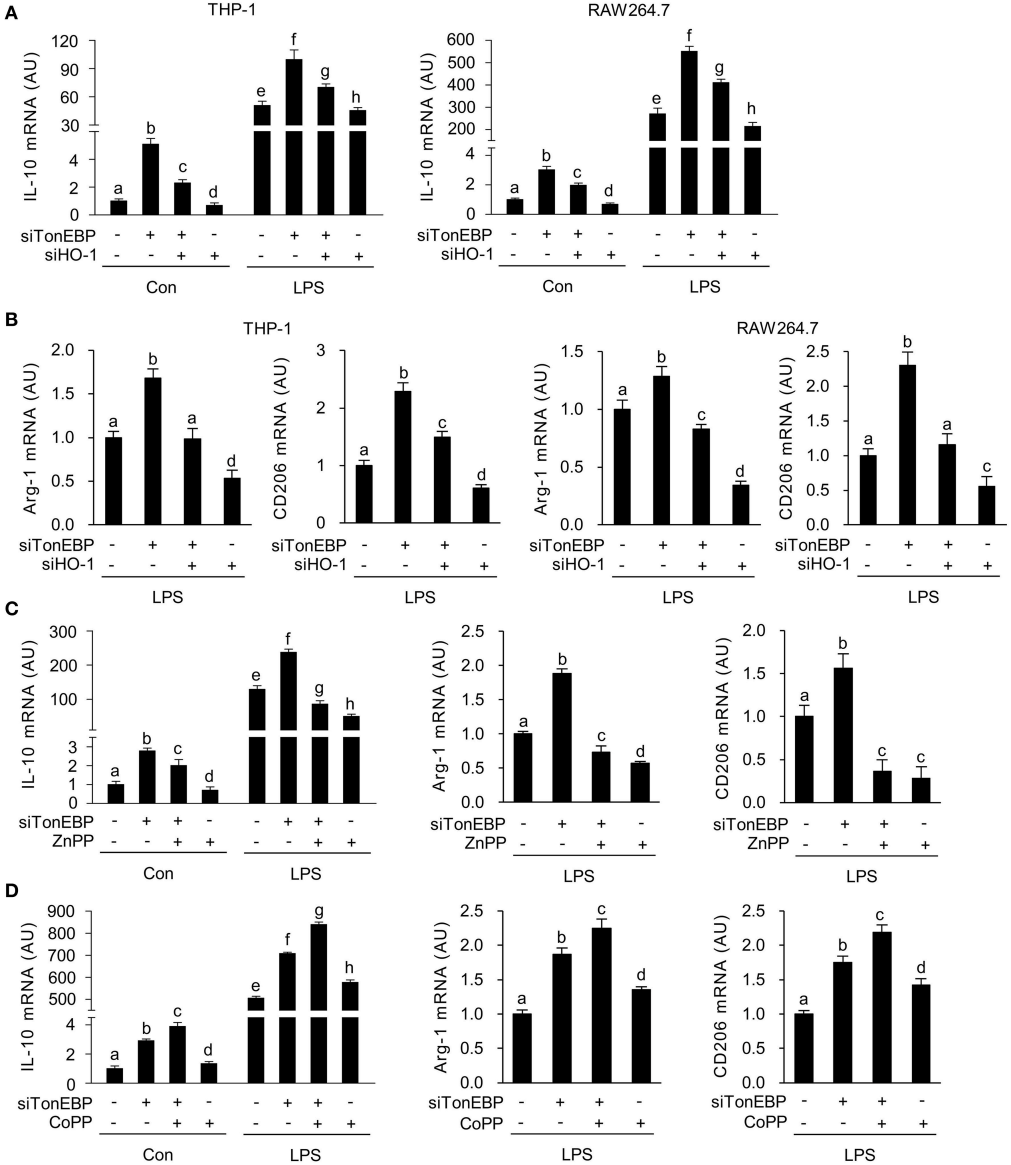
TonEBP suppresses the macrophage M2 phenotype by downregulating HO-1. **(A,B)** Differentiated THP-1 **(A)** and RAW264.7 **(B)** cells were transfected with scrambled [Scr (-)], TonEBP-targeting, or HO-targeting siRNA in the combinations indicated at the bottom of the panels for 24 h. The concentration of total siRNA was equalized by adjusting the concentration of Scr (-) siRNA. Transfected cells were then treated with LPS (100 ng/ml) for 12 h, and expression of mRNA encoding IL-10, Arg-1, and CD206 was measured by quantitative RT-PCR. **(C,D)** RAW264.7 cells transfected with Scr (-) or TonEBP-targeting siRNA were treated for 12 h with LPS in the presence of ZnPP (20 μM), CoPP (5 μM), or vehicle (-). The abundance of mRNA encoding IL-10, Arg-1, and CD206 was measured by quantitative RT-PCR. Two-way ANOVA with Tukey's *post-hoc* test was used for multiple comparisons. Different letters indicate statistical differences at *P* < 0.05. Data (mean + SD) were from three independent experiments (*n* = 3) each with more than three replicates. AU, arbitrary units.

LPS-stimulated inflammatory responses lead to expression of the anti-inflammatory cytokine interleukin-10 (IL-10) (54, 55), and M2 genes, such as arginase-1 (Arg-1) and CD206 (56); this acts as a feedback mechanism that curtails inflammatory responses. Because induction of HO-1 in macrophages promotes expression of IL-10 (57), we examined whether TonEBP knockdown-mediated induction of HO-1 played a role in the expression of IL-10. As reported previously (39), TonEBP knockdown increased expression of IL-10 mRNA in THP-1 and RAW264.7 cells under unstimulated and LPS-stimulated conditions, whereas HO-1 knockdown reduced IL10 expression (Figure 3A). The TonEBP knockdown-mediated increase in IL-10 expression in both cell types was attenuated by TonEBP/HO-1 double knockdown under unstimulated and LPS-stimulated conditions (Figure 3A). In addition, LPS-induced expression of mRNA encoding Arg-1 and CD206 increased upon TonEBP knockdown in both cell types, and TonEBP/HO-1 double knockdown attenuated the TonEBP knockdown-mediated increase in expression of Arg-1 and CD206 (Figure 3B). Furthermore, treatment of TonEBP knockdown cells with ZnPP almost completely abolished the TonEBP knockdown-mediated increase in expression of IL-10, Arg-1, and CD206 by LPS-stimulated RAW264.7 cells (Figure 3C). Reversely, treatment with CoPP promoted the TonEBP knockdown-mediated increase in expression of IL-10, Arg-1, and CD206 by LPS-stimulated RAW264.7 cells (Figure 3D). These data suggest that HO-1 mediates the stimulation of M2 genes in response to TonEBP knockdown.

In macrophages, HO-1 and IL-10 form a positive feedback loop that amplifies the anti-inflammatory response. Briefly, HO-1 promotes expression of IL-10 (58), which then feeds back to induce expression of HO-1 (59, 60). Given the finding that TonEBP knockdown increases expression of HO-1, and the results of our previous report showing that TonEBP knockdown induces the M2 phenotype by upregulating IL-10 (39), we next used siRNA to elucidate the relationship between HO-1 and IL-10. Expression of HO-1 mRNA was not affected by siRNA-mediated knockdown of IL-10 in resting and LPS-stimulated RAW264.7 cells (Supplementary Figure 3A). However, knockdown of HO-1 reduced IL-10 expression in both cell types (Supplementary Figure 3B), demonstrating that HO-1 contributes to expression of IL-10 both in resting and LPS-stimulated RAW264.7 cells.

**Figure 4:**
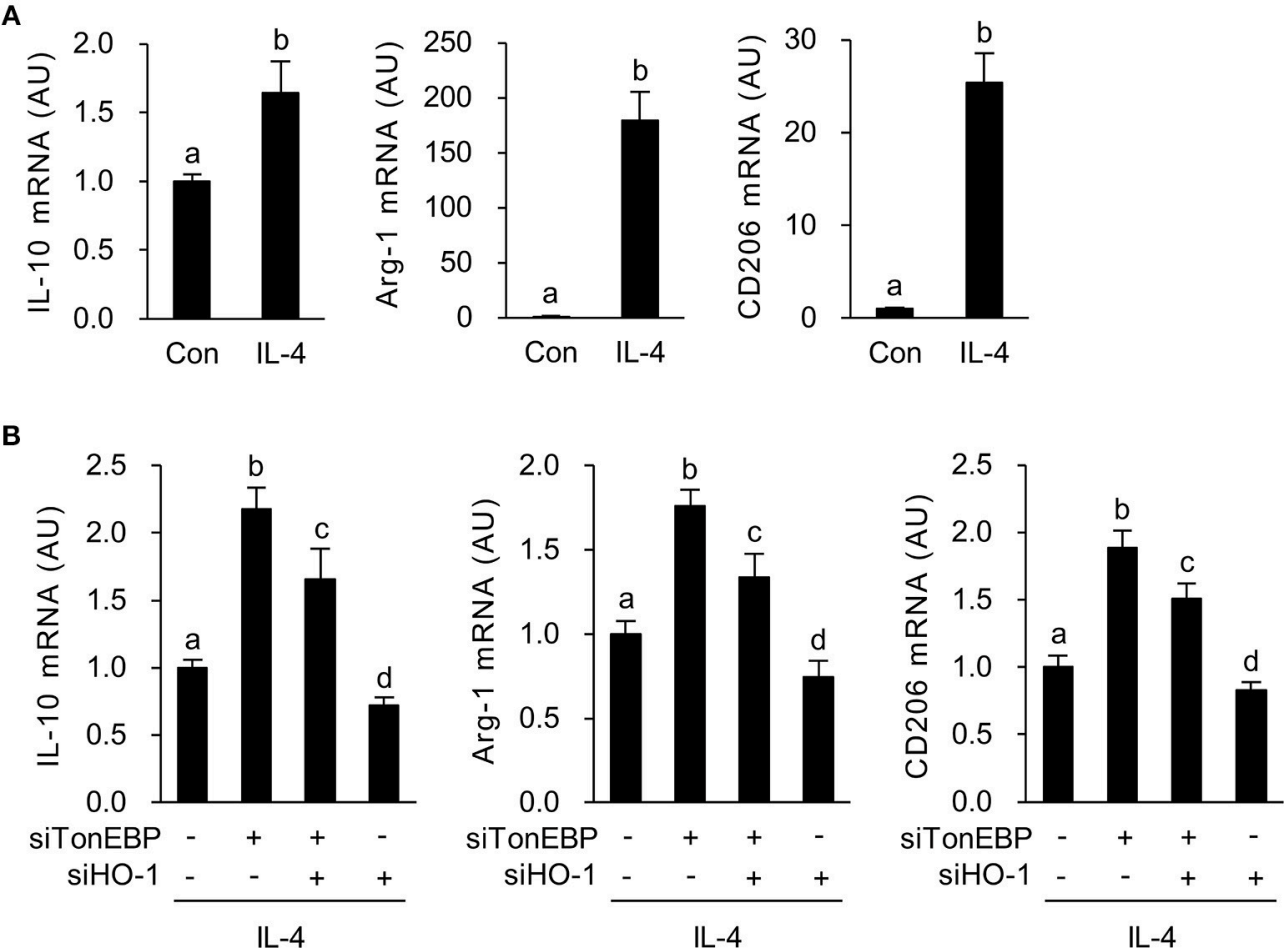
HO-1 mediates the stimulatory effects of TonEBP knockdown on the M2 phenotype. **(A)** RAW264.7 cells were treated with IL-4 (10 ng/ml) for 12 h. Expression of mRNA encoding IL-10, Arg-1, and CD206 was measured by quantitative RT-PCR. **(B)** Cells were transfected with scrambled [Scr (-)], TonEBP-targeting, or HO-targeting siRNA in the combinations indicated at the bottom of the panel for 24 h. The concentration of total siRNA was equalized by adjusting the concentration of Scr (-) siRNA. Transfected cells were then treated with IL-4 (10 ng/ml) for 12 h, and expression of mRNA encoding IL-10, Arg-1, and CD206 was measured by quantitative RT-PCR. Two-way ANOVA with Tukey's *post-hoc* test was used for multiple comparisons. Different letters indicate statistical differences at *P* < 0.05. Data (mean + SD) were from three independent experiments (*n* = 3) each with more than three replicates. AU, arbitrary units.

Next, we asked whether increased expression of HO-1 in response to TonEBP knockdown played a role in induction of the M2 phenotype in IL-4-stimulated macrophages. As previously reported (39), IL-4 induced expression of mRNA encoding IL-10, Arg-1, and CD206 in RAW264.7 cells (Figure 4A). TonEBP knockdown promoted IL-4-induced expression of mRNA encoding IL-10, Arg-1, and CD206, whereas HO-1 knockdown reduced expression of these genes in response to IL-4 (Figure 4B). The TonEBP knockdown-mediated increase in expression of mRNA encoding IL-10, Arg-1, and CD206 was suppressed by TonEBP/HO-1 double knockdown (Figure 4B). Taken together, the data in Figures 3, 4 demonstrate that suppression of M2 phenotype by TonEBP is mediated by reduced expression of HO-1.

### TonEBP Blocks Recruitment of Nrf2 to the Enhancer Region of the HO-1 Gene

**Figure 5:**
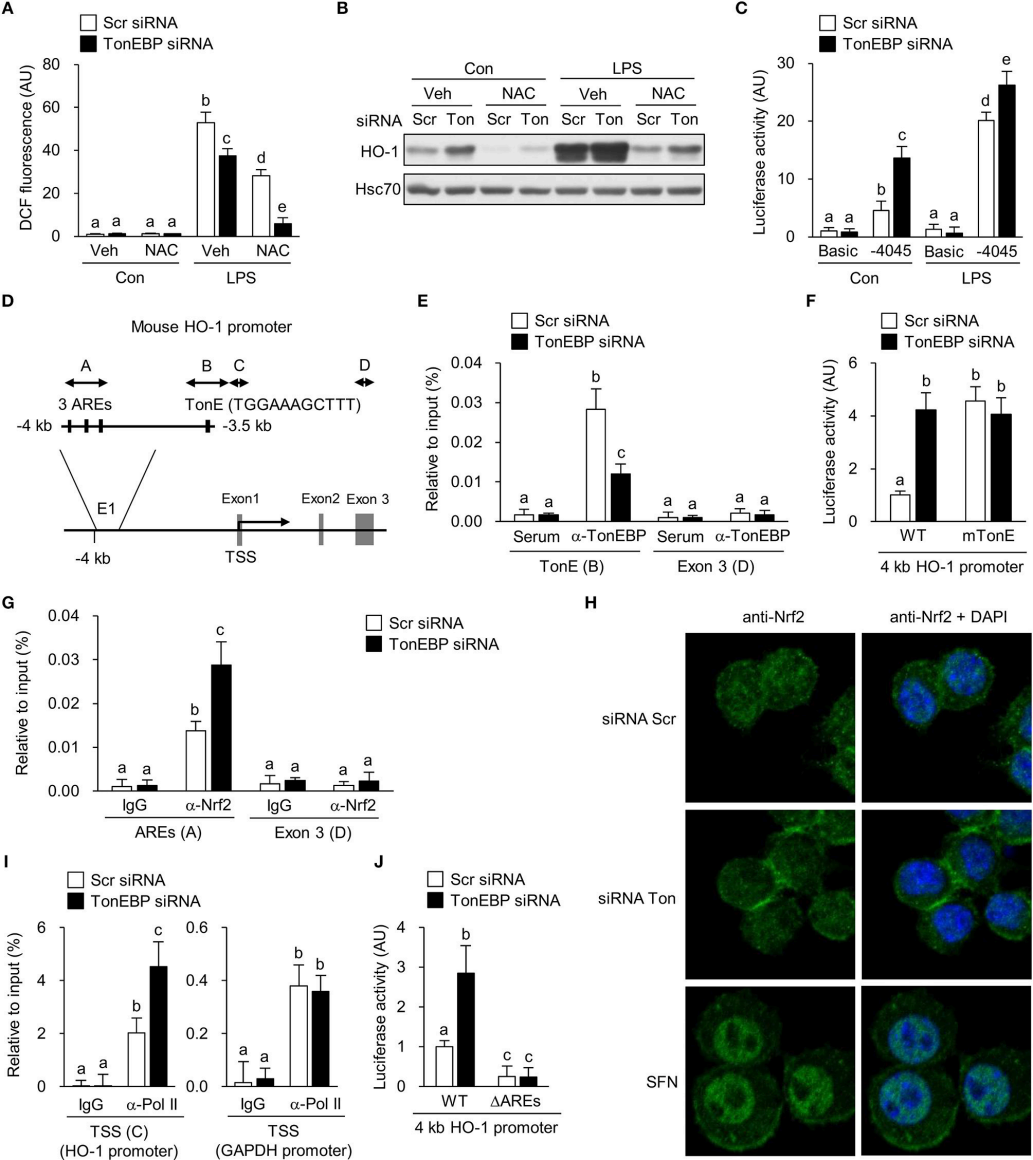
TonEBP regulates recruitment of Nrf2 to the enhancer region of the HO-1 gene. **(A–C)** RAW264.7 cells were transfected with scrambled (Scr) or TonEBP-targeting siRNA (Ton) for 24 h. Cells were pre-treated with vehicle (Veh) or NAC (10 mM) for 30 min and cultured in the presence of LPS (100 ng/ml) for 24 h. **(A)** Intracellular ROS levels were measured by DCF oxidation. **(B)** Immunoblotting to detect HO-1 and Hsc70 was performed. **(C)** The siRNA-transfected cells were transfected a second time with plasmid constructs containing a −4,045/+74 kb fragment of the mouse HO-1 promoter (−4,045/+74 pGL3) for 24 h. Luciferase activity was measured 8 h after treatment with vehicle (Con) or 100 ng/ml LPS. **(D)** Schematic representation of the mouse HO-1 gene promoter region (E1), including the AREs and TonE. **(A–D)** Indicate regions targeted by ChIP-quantitative RT-PCR. **(E)** RAW264.7 cells were transfected with Scr or TonEBP-targeting siRNA for 24 h. Immunoprecipitation was performed using an anti-TonEBP antibody or serum. Precipitated DNA, along with input DNA, was analyzed by quantitative RT-PCR using primer pairs specific for two regions of the HO-1 promoter: a proximal region covering the TonEBP binding site and exon 3 region (as a control). **(F)** The siRNA-transfected cells were transfected a second time with the −4,045/+74 promoter construct (WT) or with a mutant −4,045/+74 construct [in which the TonE site was mutated (mTonE)] and luciferase activity was measured (*n* = 4). **(G)** RAW264.7 cells were transfected with Scr or TonEBP-targeting siRNA for 24 h. ChIP assay was performed using an anti-Nrf2 antibody or IgG to detect AREs **(A)** (*n* = 3) and the exon 3 (E3) region on the HO-1 gene of RAW264.7 cells. **(H)** Confocal immunofluorescence images of Nrf2 protein. RAW264.7 cells were transfected with scrambled (Scr) or TonEBP-targeting siRNA (Ton) for 24 h. Sulforaphane (SFN) was used as a positive control of Nrf2 nuclear localization. The signals of Nrf2 protein (green) were detected using anti-Nrf2 antibody. Nuclei were counterstained with DAPI (blue). Data are representative of three independent experiments. **(I)** A ChIP assay was performed using an anti-Pol II antibody or IgG to detect the TSS **(C)** of the HO-1 promoter and the TSS region of the GAPDH promoter (as a control). **(J)** The siRNA-transfected cells were transfected a second time with the −4,045/+74 promoter construct (WT) or a mutant −4,045/+74 construct [in which the three AREs are mutated (ΔAREs)]. Luciferase activity was measured. **(A,C,E–G,I,J)** Two-way ANOVA with Tukey's *post-hoc* test was used for multiple comparisons. Different letters indicate statistical differences at *P* < 0.05. Data (mean + SD) were from three independent experiments (*n* = 3) each with more than three replicates. AU, arbitrary units.

Next, we investigated molecular mechanism underlying TonEBP-mediated regulation of the HO-1 gene. First, we examined generation of reactive oxygen species (ROS), which induce expression of the HO-1 gene (61). TonEBP knockdown did not affect ROS levels in resting macrophages for up to 48 h (Supplementary Figure 4A). Furthermore, TonEBP knockdown reduced LPS-mediated ROS generation and acted synergistically with NAC, a ROS scavenger, to reduce ROS levels further (Figure 5A). Pre-treatment of resting and LPS-stimulated cells with NAC reduced expression of HO-1 (Figure 5B) as expected. TonEBP knockdown increased expression of HO-1 in control and NAC-exposed cells, despite the lower levels of ROS accumulation (Figure 5B). These data demonstrate that depleting TonEBP induces HO-1 expression independently of ROS.

Next, we asked whether TonEBP knockdown increased transcription of HO-1. For this, we constructed a pGL3 luciferase reporter using a ~4 kb HO-1 promoter fragment containing the enhancer E1 region which is a key regulator of the HO-1 gene transcription [reviewed in Ref. (62)]. TonEBP knockdown stimulated the HO-1 promoter-driven luciferase expression in resting and LPS-stimulated RAW264.7 cells (Figure 5C). The 4 kb fragment contains one TonE sequence (a TonEBP binding sequence) near the three antioxidant response elements (AREs) that bind to nuclear factor erythroid-derived 2-like 2 (NFE2L2, Nrf2), a critical regulator of HO-1 (Figure 5D). Therefore, we asked whether TonEBP binds to the TonE site. Because TonEBP knockdown activated basal expression of HO-1 even in the absence of LPS (Figure 1), we performed a ChIP assay using resting RAW264.7 cells. Fragments of the region containing TonE were precipitated by an antibody specific for TonEBP; this precipitation was abrogated by TonEBP deficiency (Figure 5E), demonstrating that TonEBP binds to this region on chromatin. To investigate whether stimulation of the HO-1 promoter in response to TonEBP knockdown was dependent on the TonE sequence within the promoter, we constructed a mutant construct (mTonE) in which TonE was inactivated by site-directed mutagenesis. mTonE showed enhanced transcriptional activity, which was not affected by TonEBP knockdown (Figure 5F), confirming the functionality of TonE on the HO-1 promoter activity. Next, we asked whether binding of TonEBP to the TonE site affected binding of Nrf2 to the neighboring AREs. Recruitment of Nrf2 to the AREs in the enhancer E1 region was stimulated by TonEBP knockdown (Figure 5G). On the other hand, TonEBP knockdown did not affect protein expression (Supplementary Figure 4B) or nuclear translocation of Nrf2 (Figure 5H and Supplementary Figure 4C), suggesting that TonEBP directly prevents recruitment of Nrf2 to the enhancer E1 region of the HO-1 gene. Anti-Nrf2 antibody specificity was confirmed in cells transfected with Nrf2-targeting siRNA (Supplementary Figure 4D). Because recruitment of Nrf2 to the HO-1 enhancer E1 region facilitates binding of RNA polymerase II (RNA Pol II) to the human HO-1 promoter region (63), we examined RNA Pol II enrichment at the transcription start site (TSS). Recruitment of Pol II to the TSS region of the HO-1 promoter was detected in resting cells, and its binding increased in response to TonEBP knockdown (Figure 5J), consistent with elevated Nrf2 binding to the AREs. These data demonstrate that TonEBP binding reduces Nrf2 recruitment to the AREs leading to reduced Pol II binding to the promoter.

Finally, we asked whether increased expression of HO-1 upon TonEBP knockdown required Nrf2 binding to AREs. To answer the question, we deleted the three AREs from the HO-1 promoter reporter construct (Figure 5D). Deletion of AREs (ΔAREs) markedly reduced HO-1 promoter activity, which is consistent with the function of Nrf2 as a major transcriptional regulator of HO-1 [reviewed in Ref. (62)] (Figure 5K). Importantly, while wild-type HO-1 promoter-driven luciferase activity increased after TonEBP knockdown, TonEBP knockdown did not alter HO-1 promoter activity in the construct lacking AREs, demonstrating Nrf2-dependent suppression of HO-1 transcription by TonEBP.

### TonEBP-deficient M1 Macrophages Exert Paracrine Anti-inflammatory Effects

**Figure 6:**
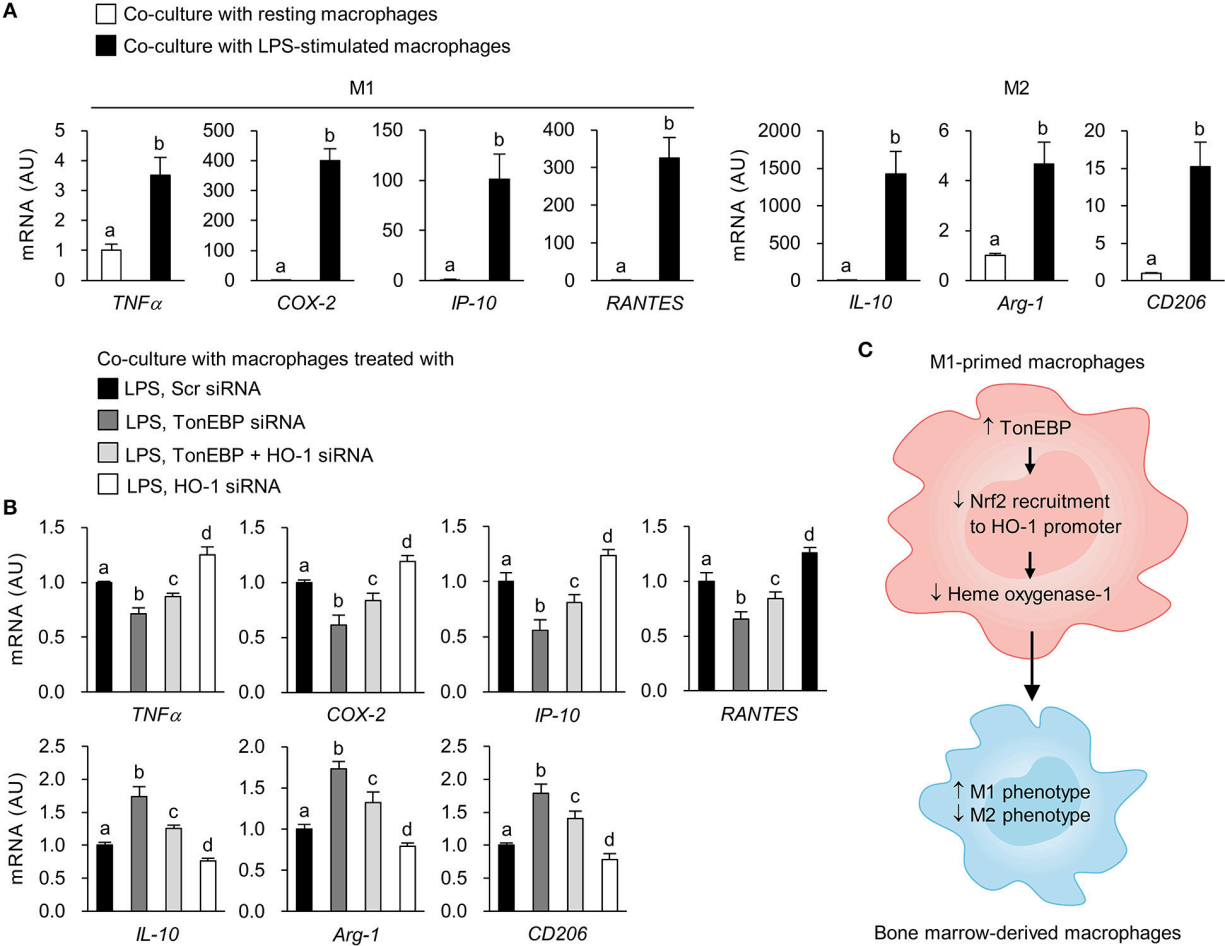
Loss of TonEBP from M1 macrophages induces paracrine anti-inflammatory effects on bone marrow-derived macrophages. **(A,B)** RAW264.7 cells were plated in transwell permeable supports, transfected with scrambled siRNA or siRNA specific for target genes for 24 h, and then treated with LPS (100 ng/ml) for 12 h. RAW264.7 cells were then added to the 6-well companion plates and co-cultured with BMDMs for 3 h (for TNFα) or 12 h (for COX-2, IP-10, RANTES, IL-10, Arg-1, and CD206) (see Supplementary Figure 5). BMDMs were collected, and expression of mRNA encoding the genes indicated at the bottom of the panel was measured by quantitative RT-PCR. Two-way ANOVA with Tukey's *post-hoc* test was used for multiple comparisons. Different letters indicate statistical differences at *P* < 0.05. Data (mean + SD) were from three independent experiments (*n* = 3) each with more than three replicates. AU, arbitrary units. **(C)** Proposed mechanism for the role of TonEBP. Increased expression of TonEBP by M1-primed macrophages suppresses HO-1 expression, leading to increased expression of M1 genes and reduced expression of M2 genes.

To examine whether TonEBP deficiency in M1-primed macrophages affects activation of resting macrophages, we conducted indirect co-culture experiments using the Transwell system. Control and TonEBP knockdown RAW264.7 cells were stimulated with LPS to induce an M1 phenotype. Then, LPS was removed prior to co-culture of M1-primed macrophages with resting BMDMs (Supplementary Figure 5). Co-culture with M1-primed macrophages induced expression of M1 (TNFα, COX-2, IP-10, RANTES) and M2 genes (IL-10, Arg-1, CD206) in BMDMs (Figure 6A). Co-culture of BMDMs with TonEBP knockdown M1 macrophages resulted in lower expression of pro-inflammatory M1 genes and higher expression of anti-inflammatory M2 genes (compared with control macrophages) (Figure 6B). However, co-culture of BMDMs with HO-1 knockdown M1 macrophages induced expression of M1 genes and attenuated expression of M2 genes (compared with controls) (Figure 6B). Moreover, double knockdown of TonEBP and HO-1 reduced the effects of TonEBP knockdown on activation of BMDMs (Figure 6B), confirming that M1 macrophages push surrounding resting macrophages into M1 phenotype, and that TonEBP-mediated priming toward M1 macrophages is driven by downregulation of HO-1 expression.

## Discussion

Dynamic changes in the functional phenotype of macrophages are associated with pathogenesis of inflammatory diseases (5–7). TonEBP primes macrophages toward an M1 phenotype, which has pro-inflammatory properties. TonEBP does this by promoting expression of pro-inflammatory genes via interaction with NF-κB (36) and by binding directly to the promoter (37, 64). In addition, TonEBP suppresses expression of the anti-inflammatory cytokine IL-10 by limiting chromatin access to the promoter (37). The pro-inflammatory function of TonEBP suggests that inhibiting its expression or activation could suppress inflammatory responses. Indeed, TonEBP haplo-deficient and myeloid-specific TonEBP knockout mice are effectively protected from inflammatory diseases. TonEBP haplo-insufficiency in a mouse model of rheumatoid arthritis almost completely prevented pannus formation and cartilage destruction, which was related to the reduced survival of macrophages (16, 34). Also, formation of atherosclerotic lesions in *ApoE*^−/−^ mice fed a high fat diet is reduced when mice are TonEBP haplo-deficient; this reduction is dependent on TonEBP depletion from macrophages (32). In a mouse model of diabetic nephropathy (DN), TonEBP haplo-deficiency is associated with reduced activation of macrophages by hyperglycemia, with fewer macrophages in the kidney, with lower renal expression of pro-inflammatory genes, and with attenuated DN (35). Moreover, increased activity of TonEBP in monocytes is associated with early DN in humans (65). A recent study shows that TonEBP promotes hepatocellular carcinogenesis, recurrence, and metastasis in patients with hepatocellular carcinoma (HCC) and in mouse models of HCC (36).

Here, we identified a novel function of TonEBP as a potent suppressor of HO-1 expression both in human and murine macrophages. The role of TonEBP in suppressing expression of HO-1 is important given the well-established immunosuppressive activity of HO-1. HO-1-deficient mice show increased oxidative stress, a tendency toward pro-inflammatory responses (15, 21), and increased susceptibility to sepsis (17). Phenotypical alterations in human cases of genetic HO-1 deficiency are similar to those observed in HO-1 knockout mice (19, 20). Furthermore, increased HO-1 expression in macrophages leads to a reduced capacity for foam cell formation (a potent anti-inflammatory and tissue regenerative function) and thereby suppresses atherosclerosis (66). Activation of HO-1 ameliorates renal damage in STZ-induced DN in rats through anti-inflammatory and antioxidant mechanisms (44). Genetic and pharmacological induction of HO-1 expression in synovial cells from RA patients reduces expression of pro-inflammatory genes (67). In this regard, TonEBP depletion-driven immunosuppression resembles the immunosuppressive effects of HO-1. Importantly, we show here that depleting TonEBP promotes expression of HO-1 even under basal conditions. This finding is of great interest because increasing of evidence suggests basal HO-1 levels are more important in the protection against inflammation and oxidative stress than the degree of upregulation of HO-1 following injury (21, 68, 69). Thus, this study provides an opportunity to further our understanding of the role of TonEBP during polarization, and on the functions, of macrophages. As such, it may facilitate design of new regimens that prevent inflammatory diseases.

Expression of HO-1 is regulated primarily at the transcriptional level, and distinct DNA sequence-dependent enhancer regions in the upstream regulatory regions of the HO-1 promoter mediate basal and inducible HO-1 gene expression in different species [reviewed in Refs. (14, 62)]. One major *cis*-acting DNA sequence element in the enhancers is called stress-responsive element which contains AREs. Cognate transcription factor for AREs is Nrf2, a Cap“n”collar/basic-leucine zipper transcription factor (70). Under basal conditions, Keap1 forms a complex with Nrf2 and limits its nuclear translocation (71). When cells are exposed to inducing stimuli, such as endotoxin, heme, and oxidants, Nrf2 dissociates from Keap1, translocates to the nucleus, and binds to the AREs (72). Here, we suggest a new regulatory mechanism for Nrf2-mediated HO-1 induction in macrophages: downregulation of TonEBP stimulates HO-1 expression by recruitment of Nrf2 to the enhancer region of the HO-1 gene without affecting nuclear translocation of Nrf2. The TonEBP depletion-mediated increase in HO-1 expression attenuates polarization of macrophages toward the pro-inflammatory M1 phenotype while enhancing M2 polarization (Figure 6C). Identification of HO-1 as a downstream target of TonEBP provides an exciting opportunity for the design and development of novel therapeutic approaches that resolve chronic inflammation associated with inflammatory diseases.

## Ethics Statement

All experimental protocols were approved by the Institutional Animal Care and Use Committee of the Ulsan National Institute of Science and Technology (UNISTACUC-12-15-A).

## Author Contributions

EY, SC, and HMK designed the experiments and wrote the manuscript. EY, SC, HL, BY, JL, CL, HJK, GJ, HP, SL, and WL performed the experiments. EY, SC, and HMK analyzed data.

### Conflict of Interest Statement

The authors declare that the research was conducted in the absence of any commercial or financial relationships that could be construed as a potential conflict of interest.

## Supplementary Material

Supplementary Figure 1 TonEBP suppresses expression of HO-1 in macrophages. **(A)** PM and BMDM obtained from TonEBP^+/+^ or TonEBP^+/Δ^ mice were cultured in normal glucose (5.5 mM) and treated for 6 h with vehicle (Con) or LPS (100 ng/ml). Quantitative RT-PCR was performed for mRNA for TonEBP and HO-1. **(B)** RAW264.7 cells were transfected with scrambled siRNA or siRNA targeting TonEBP. The transfected cells were cultured in normal glucose (5.5 mM), high glucose (25 mM), or 5.5 mM glucose +19.5 mM mannitol (osmotic control for high glucose) for 24 h and then treated with LPS (100 ng/ml) for 6 h. Quantitative RT-PCR was performed to measure expression of mRNA encoding TonEBP and HO-1. **(C)** RAW264.7 cells were infected with an empty control virus (Ad-EV) or an adenovirus carrying the human TonEBP gene (Ad-TonEBP) at an MOI of 20, 50, 100 or 200 for 24 h. Control cells were mock-infected with PBS. Cytotoxicity was assessed by release of LDH into the culture media after 24 h of infection. **(D)** Differentiated THP-1 cells were transfected with scrambled [Scr (-)] or two siRNAs (hTon #1 or hTon #2) targeting different regions of human TonEBP mRNA for 24 h. Immunoblotting to detect TonEBP, HO-1 and Hsc70 was performed. Data are representative of three independent experiments. **(A–C)** Two-way ANOVA with Tukey's *post-hoc* test was used for multiple comparisons. Different letters indicate statistical differences at *P* < 0.05. Data (mean + SD) were from three independent experiments (*n* = 3) each with more than three replicates. AU, arbitrary units.

Supplementary Figure 2 The expression of TonEBP and HO-1 by gene-targeting siRNAs or modulators of HO-1 in THP-1 and RAW264.7 cells. **(A–D)** Differentiated THP-1 **(A)** and RAW264.7 **(B–D)** cells were transfected with scrambled [Scr (-)], TonEBP-targeting, and HO-targeting siRNA for 24 h in the combinations indicated at the bottom of the panels. The concentration of total siRNA was equalized by adjusting the concentration of Scr (-) siRNA. **(A,B)** Transfected cells were then treated with LPS (100 ng/ml) for 3 h. Expression of TonEBP and HO-1 mRNA was measured by quantitative RT-PCR. **(C,D)** RAW264.7 cells transfected with Scr (-) or TonEBP-targeting siRNA were treated for 3 h with LPS in the presence of ZnPP (20 μM), CoPP (5 μM), or vehicle (-). Expression of mRNA was measured by quantitative RT-PCR. **(E)** RAW264.7 cells transfected with scrambled [Scr (-)] or TonEBP-targeting siRNA (Ton) for 24 h were treated for 18 h with LPS in the presence of vehicle (-) or CoPP (1 or 5 μM). Immunoblotting was performed to detect HO-1 and Hsc70. Data are representative of three independent experiments. **(A–D)** Two-way ANOVA with Tukey's *post-hoc* test was used for multiple comparisons. Different letters indicate statistical differences at *P* < 0.05. Data (mean + SD) were from three independent experiments (*n* = 3) each with more than three replicates. AU, arbitrary units.

Supplementary Figure 3 HO-1 contributes to expression of IL-10 in macrophages. RAW264.7 cells were transfected with scrambled (Scr), or IL-10- or HO-1-targeting siRNA for 24 h, followed by treatment with vehicle (Con) or 100 ng/ml LPS for 6 h. Quantitative RT-PCR was performed to measure expression of mRNA encoding HO-1 **(A)** and IL-10 **(B)**. Two-way ANOVA with Tukey's *post-hoc* test was used for multiple comparisons. Different letters indicate statistical differences at *P* < 0.05. Data (mean + SD) were from three independent experiments (*n* = 3) each with more than three replicates. AU, arbitrary units.

Supplementary Figure 4 TonEBP knockdown does not affect ROS levels and nuclear translocation of Nrf2. **(A–D)** RAW264.7 cells were transfected with scrambled (Scr) or TonEBP-targeting siRNA (Ton) for 24 h. **(A)** Cells were pre-treated with vehicle (Veh) or NAC (10 mM) for 30 min and then cultured for 24 or 48 h. Intracellular ROS levels were determined by DCF oxidation. Two-way ANOVA with Tukey's *post-hoc* test was used for multiple comparisons. Data (mean + SD) were from three independent experiments (*n* = 3) each with more than three replicates. AU: arbitrary units. **(B,D)** Cells were treated with vehicle (Veh) or LPS (100 ng/ml) for 1 h and harvested, and cell nuclei and cytoplasm were separated using a Nuclear and Cytoplasmic extraction kit (Pierce) according to the manufacturer's instructions. **(B)** Immunoblotting was performed to detect Nrf2, p65, TonEBP, and Hsc70 (control) in whole cell lysates derived from cells transfected with Scr or TonEBP-targeting siRNA). **(C)** Anti-Nrf2 antibody specificity was confirmed in cells transfected with Nrf2-targeting siRNA. **(D)** Immunoblotting was performed to detect Nrf2, p65, and TonEBP in the nuclear or cytosolic fractions. The nuclear fraction was confirmed by detection of Lamin B. Data are representative of three independent experiments.

Supplementary Figure 5 A diagram of the system used to co-culture mouse RAW264.7 cells and bone marrow-derived macrophages (BMDMs). Progenitors in the bone marrow were differentiated into macrophages by exposure to M-CSF for 9 days and then plated into 6-well plates. RAW264.7 cells were plated into culture inserts, transfected with scrambled siRNA or siRNA specific for target genes for 24 h, and then treated with LPS (100 ng/ml) for 12 h. The cells were then added to the 6-well companion plates and co-cultured with BMDMs for 3 or 12 h. At the end point of the experiment, BMDMs were collected and used in a gene expression assay to assess the paracrine effects of macrophages.
