# Supplementary material for: TonEBP Suppresses the HO-1 Gene by Blocking Recruitment of Nrf2 to Its Promoter
Source: Front Immunol. 2019 Apr 18;10:850. doi: 10.3389/fimmu.2019.00850 (PMC6482272; doi:10.3389/fimmu.2019.00850)
Supplement: Supplementary file 1 [file Table_1.pdf]

Supplementary Table 1. Primers used in qPCR for gene expression analysis and ChIP assay

Primers for gene expression analysis

Gene	Forward primer (5'-3')	Reverse primer (5'-3')
Mouse *TonEBP*	AAGCAGCCACCACCAAACATGA	AAATTGCATGGGCTGCTGCT
Mouse *HO-1*	CTCCCTGTGTTTCCTTTCTCTC	GCTGCTGGTTTCAAAGTTCAG
Mouse *TNFa*	TGGGACAGTGACCTGGACTGT	TTCGGAAAGCCCATTTGAGT
Mouse *COX-2*	CGGACTGGATTCTATGGTGAAA	CTTGAAGTGGGTCAGGATGTAG
Mouse *IP-10*	AGTAACTGCCGAAGCAAGAA	GCACCTCCACATAGCTTACA
Mouse *RANTES*	CCAATCTTGCAGTCGTGTTTG	ACCCTCTATCCTAGCTCATCTC
Mouse *IL-10*	TTGAATTCCCTGGGTGAGAAG	TCCACTGCCTTGCTCTTATTT
Mouse *Arginase-1*	CTCCAAGCCAAAGTCCTTAGAG	AGGAGCTGTCATTAGGGACATC
Mouse *CD206*	TCTTTTACGAGAAGTTGGGGTCAG	ATCATTCCGTTCACCAGAGGG
Mouse *Cyclophilin A*	CTGCTGTCTTTGGAACTTTGTCTG	CAGCCATGGTCAACCCCACCG
Human *TonEBP*	AAGCAGCCACCACCAAACATGA	AAATTGCATGGGCTGCTGCT
Human *HO-1*	ATGGCCTCCCTGTACCACATC	TGTTGCGCTCAATCTCCTCCT
Human *TNFa*	AGAGGGAGAGAAGCAACTACA	GGGTCAGTATGTGAGAGGAAGA
Human *COX-2*	CGGACTGGATTCTATGGTGAAA	CTTGAAGTGGGTCAGGATGTAG
Human *IP-10*	GTAATAACTCTACCCTGGCACTATAA	GATGGGAAAGGTGAGGGAAATA
Human *RANTES*	TGCCCACATCAAGGAGTATTT	GATGTACTCCCGAACCCATTT
Human *IL-10*	TTTCCCTGACCTCCCTCTAA	5’CGAGACACTGGAAGGTGAATTA
Human *Arginase-1*	CAGGGCTACTCTCAGGATTAGA	CCGAAACAAGCCAAGGTTATTG
Human *CD206*	GGACGTGGCTGTGGATAAAT	ACCCAGAAGACGCATGTAAAG
Human *Cyclophilin A*	TTCATCTGCACTGCCAAGAC	TCGAGTTGTCCACAGTCAGC

Primers for ChIP assay

Gene		Forward primer (5'-3')	Reverse primer (5'-3')
Mouse *HO-1*	A (AREs)	CCACAGGAGCTGAACTTTGT	TGAGGCTGAGGGAACAG
	B (TonE)	TACAGAAGCTAGCAGTTTCATCC	CCTAAGAAAGGAGTCAGCCATAA
	C (TSS)	CGTGACCCGCGTACTTAAA	CTGGTTGTATGCGGAAACTCT
	D (Exon 3)	TGATGGCTTCCTTGTACCATATC	AGCTCCTCAGGGAAGTAGAG
Mouse *GAPDH*	TSS	GGGTTCCTATAAATACGGACTGC	CGAACTCGAGGAGAGCCATC
